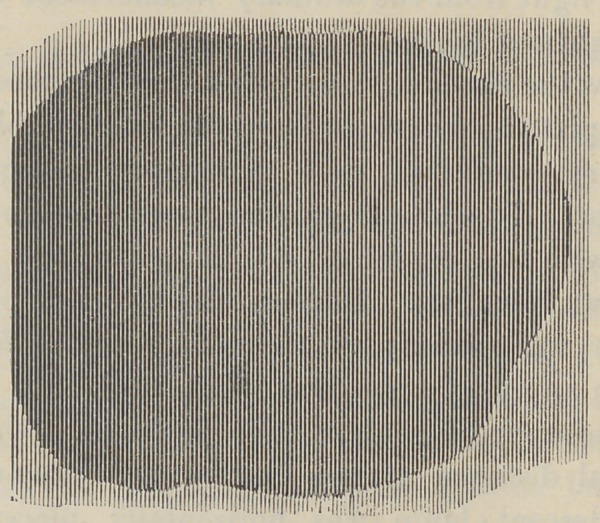# Shadowgraphs without the Crooke’s Tubes

**Published:** 1896-03

**Authors:** John L. Gish

**Affiliations:** Jackson, Mich.


### Shadowgraphs without the Crooke's Tubes.

BY JOHN L. GISH, M.D., D.D.S., JACKSON, MICH.

Since the discovery of the X rays by Prof. Roentgen, I have been deeply interested in the subject of shadowgraphs, and have accomplished something entirely new therewith. It is always good law to produce results in the most simple way; hence, after reviewing the subject of shadowgraphs, and how produced, it soon occurred that the Leyden jars, the Crooke's tubes, the Geissler tubes, etc., actuated by the static machine, or the Ruhmkorff coil, and a plant of batteries and other costly apparatus were not necessary for success in this direction, and that one could produce shadowgraphs by means of the X rays, from the common sources of light which surround us on every hand. It is this principle of making shadowgraphs by means of the X rays, from other sources than the Crooke's tube and its necessary complicated apparatus, that I have succeeded in establishing by producing the required results on photographic plates, covered by heavy felt, layers of paper, thick, heavy, cardboard, etc., taking the source of light from the ordinary incandescent lamps which are used to light our homes.

This is how the shadowgraphs below were produced: Into the photographic plate-holder were placed two sensitive plates, which, under ordinary conditions, were thoroughly protected from all sources of light. To add still greater protection the plate-holder was covered with two layers of manila paper, and then a layer of thick, heavy, blue felt. Between the layers of paper and felt a silver dollar was placed; on the outside of the felt a piece of plate glass, in an upright position was placed, thus making the two objects occupy different relative positions. Above all, and within a focal distance of two inches, an ordinary 16-candle power incandescent lamp was horizontally placed; then, in order to confine the rays and protect from any external influences, the lamp and all were covered with many layers of cloth and paper. All being in readiness the light was turned on, and an exposure of fifty minutes was allowed. At the end of this time, the plate-holder was removed and the negatives developed, so that they could be brought to the open air and thoroughly examined.

While the outlines of the shadowgraph on these plates are not as sharply defined as I would like, yet that imperfection can be corrected by proper manipulation of the light, and it does not alter the scientific principle established.

Both of the plates were acted upon at the same time. Plate No. 1 has the images quite defined, but Plate No. 2, while it shows but little to the eye of the public, is more interesting from a scientific point of view, and one reason why it is so, is as follows:

Before Plate No. 2 could be acted upon, in the smallest possible degree, the X rays had to penetrate the heavy felt, nearly one-eighth of an inch thick, two layers of paper, one layer of card-board one-eighth of an inch thick, through sensitive Plate No. 1, then through another layer of cardboard one-fourth of an inch thick to the sensitive Plate No. 2, producing a molecular change, or a rearrangement of the conditions therein, and all from so common a source of light as an incandescent lamp.

" Beyond the satisfaction of having produced shadowgraphs, by other ways than those put forth by Prof. Roentgen, we may ask, what is there in it for the public?" " The answer to this question is two-fold." " In the first place, it proves that the source of the X rays is not confined to the Crooke's tube and its accompaniments. But that we may obtain the mysterious something from the incandescent lamp, and reasoning from analogy, we have the same energetic, deep penetrating rays from the greatest of all sources of light, namely, the sun. I think also that I might add another source of supply, to come from the electric arc light, but I am not so sure about that as yet. The second part of this question concerns the public more deeply, for it comes under the domain of health and disease. It is much beyond the limit of this article to treat the subject in full, but I will make a suggestion or two and then close. For the last five or six years I have devoted as much time as I could, aside from my present specialty, to the study of electricity and its applications to the human body in overcoming certain forms of disease, and the one subject of electric-light bath, as a substitute for the sun bath, has commanded no small amount of my time and attention; what I have been able to do myself, coupled with the results of other men, has proved the value of such a bath. I must confess that such baths have been used more or less empirically; we simply knew that good results did come, but we did not know why, but now the shadowgraphs on the sensitive plates will undoubtedly help us to analyze this special class of work; it will make us better masters of the subject, and allow us to limit and increase our lines of action; hence, the public benefit."

" With regard to shadowgraphing the different internal structures of the body and locating diseased portions and foreign substances?" "There is not any question, from what has been accomplished, that with plenty of power to produce the X rays, and handled in the proper manner, but that we can project them clear through the human body. But I believe to locate foreign substances, and to outline diseased portions of our body, direct illumination of the parts or structures will give far better results, just as you can look through the finger."

With a one-half candle power-electric lamp, placed just in the proper position, and should there be any diseased portion or foreign substance in the finger, even an unexperienced eye could locate it at once.

## Figures and Tables

**Figure f1:**
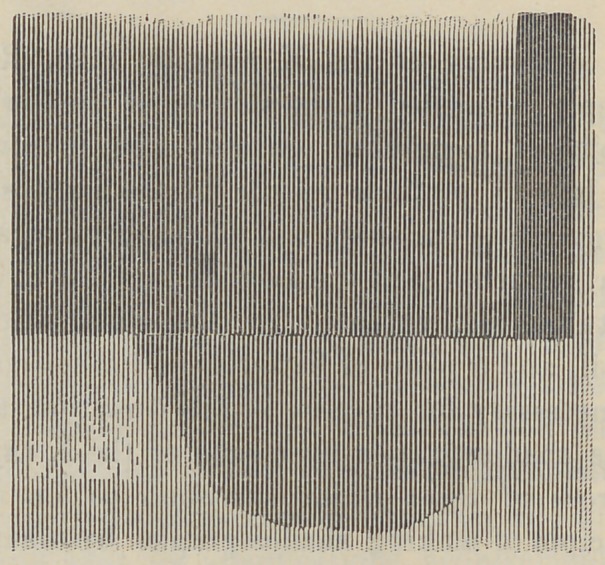


**Figure f2:**